# Visualized Analysis of Global Studies on Cervical Spondylosis Surgery: A Bibliometric Study Based on Web of Science Database and VOSviewer

**DOI:** 10.1007/s43465-021-00581-5

**Published:** 2022-02-16

**Authors:** Tianji Huang, Weiyang Zhong, Chao Lu, Chunyang Zhang, Zhongqi Deng, Runtao Zhou, Zenghui Zhao, Xiaoji Luo

**Affiliations:** Department of Orthopedic Surgery, The First Affiliated Hospital of Chongqing Medical University, Chongqing, 400016 People’s Republic of China

**Keywords:** Cervical spondylosis, Spine, Surgery, Bibliometric, VOSviewer

## Abstract

**Purpose:**

This study used multiple type of bibliometric analysis for identifying and summarizing the publications regarding cervical spondylosis surgery, for clarifying the history of this field, predicting the future hotspots of this field and improving communication among researchers.

**Methods:**

Publications from Web of Science database between 1900 and 2019 were downloaded and analyzed by Excel 2016 and VOSviewer. Bibliometric maps of co-citations and maps of co-occurrence of keywords are constructed by VOSviewer software.

**Results:**

A total of 2110 publications were searched from Web of Science. The total sum of times cited is 40448 with the average citation per publication of 19.17 times. USA published most papers (652, 30.9%). The most productive organizations is University of Toronto (96 publications). *Spine* (308 publications) published the most publications in this field. In co-citations of references analysis, four clusters of references are constructed by VOSviewer. In co-occurrence of keywords analysis, three clusters of keywords are constructed by VOSviewer. The latest keyword “degenerative cervical myelopathy” appeared in 2017 in 42 papers. Other relatively new keywords include “surgical outcomes”, “association”, “sagittal alignment”, “prognostic-factors” that appeared in 2016 in 33, 31, 34 and 37 papers respectively.

**Conclusion:**

USA dominates the research regarding cervical spondylosis surgery. University of Toronto is the most productive organization in this field. *Spine, European Spine Journal* and *Journal of Neurosurgery Spine* are the top three productive journals on publications of cervical spondylosis surgery. “Degenerative cervical myelopathy”, “surgical outcomes”, “association”, “sagittal alignment” and “prognostic-factors” may be the new research hotspots in this field.

## Introduction

Cervical spondylosis is caused by arthritic changes in the osseocartilaginous components of the cervical spine, characterized by neck pain, radiculopathy, or/and myelopathy, that resulted from compression of the spinal cord or/and nerve roots [1]. Population-based studies found that about 80–90% people by the age of 50 years have disc degeneration changes shown on magnetic resonance imaging [2, 3], while symptomatic radiculopathy (about 83 per 100,000 persons) and myelopathy (about 4 per 100,000 persons) are much less [4, 5]. Conservative treatments such as oral analgesics, epidural glucocorticoid injections, cervical traction and physical therapy are effective in most patients with cervical radiculopathy, while a few high-quality clinical trials are still lacking. Surgical treatment should be considered for cervical myelopathy due to the progressive natural history of spinal cord compression in most patients [6]. Surgical treatment is recommended with consensus in patients with symptoms ranged from moderate to severe neurologic deficits, while the best timing for surgical intervention remains unclear [1]. Three common surgical approaches exist for cervical radiculopathy or/and cervical myelopathy include anterior, posterior and anteroposterior approaches. Each approach has its advantages and disadvantages, and should be considered case by case depending on the surgeon’s experience [7, 8]. Surgical outcomes between artificial disc replacement and fusion remain controversial [9, 10].

Bibliometrics are used for assessing the studies from different countries/regions, different institutes, or/and different authors using total number of publications, impact factor, H-index, total citation number, average citation and so on which could be also helpful for the treatment of certain diseases. Bibliometrics are helpful for clarifying the history of certain field, predicting the future hotspots of certain field, improving communication among researchers. Many medicine fields have used bibliometric analysis include surgery [11], urinary surgery [12], hematological disease [13], endocrinology [14] and physical therapy [15] for further understanding of the certain field.

Clustering scientific publications is an important problem in bibliometric research. As a free java software, VOSviewer could be used to construct and visualize bibliometric networks include countries/regions, institutes, or researchers based on co-citation, bibliographic coupling, co-authorship relations or co-occurrence of keywords extracted from scientific publication [16].

There are few bibliometric studies related to spine disease which utilized this powerful software had been published [17–20]. In recent years, publications regarding cervical spondylosis surgery are growing rapidly, while many controversial concepts are remained in the treatment option of this disease. Analyzing the publications in this surgical field is necessary. To our knowledge, this is the first study that used VOSviewer as a main tool to analyze publications regarding cervical spondylosis surgery.

## Materials and Methods

### Data Source and Search Method

Web of Science core collection database which included databases of SCI-EXPANDED, SSCI, A&HCI, CPCI-S, CPCI-SSH, BKCI-S, BKCI-SSH, ESCI, CCR-EXPANDED and IC was used to search for publications related to cervical spondylosis surgery. In the present study, the search strategy was used as: theme = (surgery AND ((cervical spondylosis) OR (cervical spondylotic radiculopathy) OR (cervical spondylotic myelopathy))). The document type was refined as article or review and the timespan was set from 1900 to 2019.

### Data Extraction

The data were downloaded from Web of Science. Microsoft Excel 2016 and VOSviewer software were used to reserve and analyze the data.

The following information were recorded: publication year, publication number, publication country/region, publication organization, journal title, author, sum of times cited and H-index. H-index could be used to evaluate a scholar’s contribution both quantitatively and qualitatively. Furthermore, H-index could be extended to assess contributions of an organization, a journal or a country/region.

### Data Analysis

Bibliometric information including publication year, publication number, publication country/region, publication organization, journal title, author, sum of times cited and H-index was analyzed by the Microsoft Excel 2016 and intrinsic function of Web of Science database both quantitatively and qualitatively.

VOSviewer, a Java software, could be downloaded and used freely, which is used to create maps from bibliographic data and to visualize and dig the intrinsic meaning of the maps. In the present study, VOSviewer was used for visualizing and analyzing co-citation of references, co-citation of authors and co-occurrence of keywords.

## Results

### General Information of Publications Regarding Cervical Spondylosis Surgery

A total of 2196 publications were found from Web of Science database. Document types of 2110 publications were refined as article or review. The total number of times cited is 40448 (26,468 without self-citations). The average citation of all the publications is 19.17 times. The H-index of all the publications on cervical spondylosis surgery is 87.

### Publication Year and Relative Research Interest

**Fig. 1:**
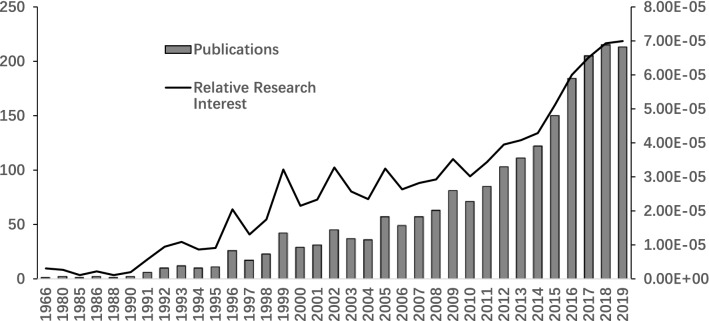
Number of studies published each year and relative research interest

The publication years of studies regarding cervical spondylosis surgery range from 1966 to 2019. The year 2018 ranked first as the most productive year with 215 publications regarding cervical spondylosis surgery. The year 2019 with 213 papers ranked second and the year 2017 with 205 papers ranked third (Fig. 1). The year 1991 and before only published less than ten papers each year.

### Countries/Regions of Publications

**Fig. 2:**
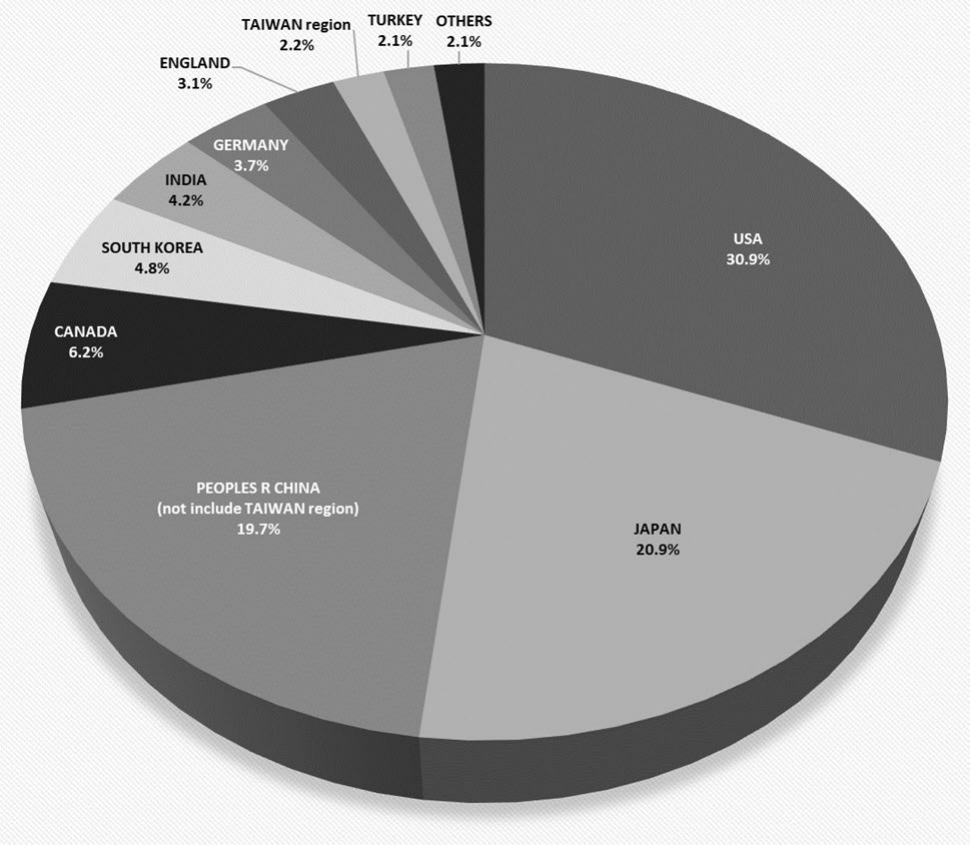
Distribution and percentage of countries/regions of publications

**Fig. 3:**
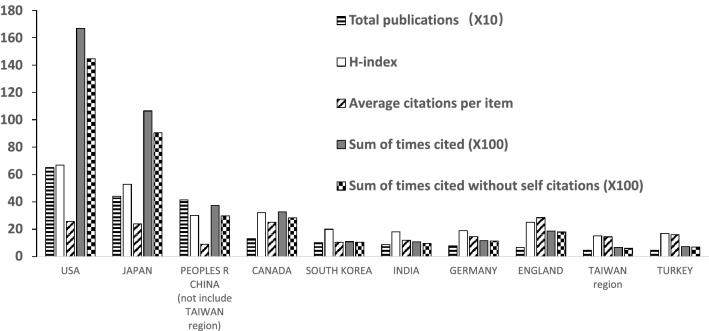
The total publications, H-index, average citations and sum of times cited of some productive countries/regions

USA published most papers (652, 30.9%), followed by Japan (442, 20.9%) and China (not include Taiwan region) (415, 19.7%) (Fig. 2). These top three countries/regions account for more than 70% of all the publications regarding cervical spondylosis surgery. Studies from USA were cited 16,682 times, ranked first of all the countries/regions, followed by Japan (10,642 times) and China (not include Taiwan region) (3738 times). The top three H-index are USA (67), Japan (53) and Canada (32), followed by China (not include Taiwan region) (30). The average citations of all the studies of England ranked first (28.46), and USA (25.59) and Canada (25.07) ranked second and third, respectively. The publication number of England ranked only eighth; however, the average citations of it ranked first of all the countries/regions. The publication number and H-index of China (not include Taiwan region) ranked third and fourth, respectively, of all the countries/regions, while the average citations of it ranked only tenth among the top ten productive countries/regions. The publications amount of Japan and China (not include Taiwan region) are almost the same; however, the total citation number of publications of Japan is almost three times as that of China (not include Taiwan region). The publications number of South Korea is about 1.5 times than that of England, while the total citation number of publications of England is almost twice as that of South Korea (Fig. 3).

### Organizations of Publications

**Fig. 4:**
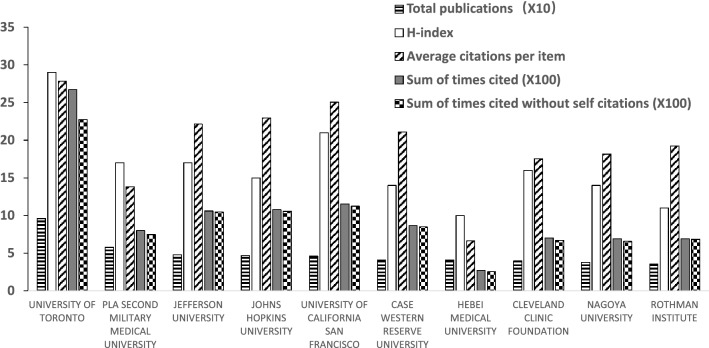
The total publications, H-index, average citations and sum of times cited of some productive organizations

The top three productive organizations regarding publications of cervical spondylosis surgery are University of Toronto (96 publications), PLA Second Military Medical University (58 publications) and Jefferson University (48 publications) (University Health Network Toronto and University of California System are not included because of duplication). Total sum of times cited of publications from University of Toronto ranked first (2671 times), followed by that from University of California San Francisco (1152 times) and Johns Hopkins University (1079 times). H-index of University of Toronto ranked first (29), followed by that of University of California San Francisco (21), Jefferson University (17) and PLA Second Military Medical University (17). The average citations of University of Toronto ranked first (27.82), and University of California San Francisco (25.04) and Johns Hopkins University (22.96) ranked second and third, respectively. PLA Second Military Medical University ranked the second most productive organizations, while the average citations only ranked ninth of all the top ten productive organizations. Case Western Reserve University and Hebei Medical University published the same number of studies regarding cervical spondylosis surgery, while the total sum of times cited of papers of Case Western Reserve University is more than three times as that of Hebei Medical University (Fig. 4).

### Journals of Publications

**Fig. 5:**
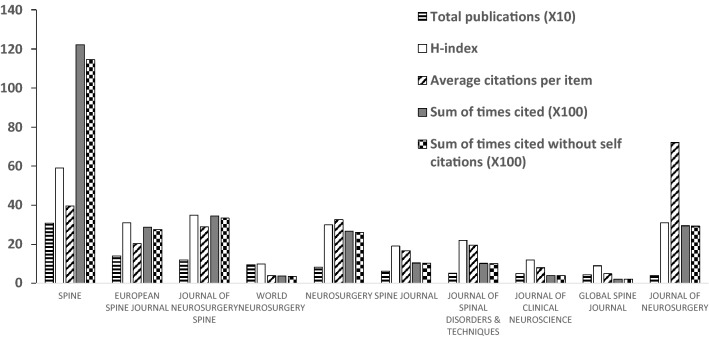
The total publications, H-index, average citations and sum of times cited of some productive journals

There are three journals that published more than 100 publications. *Spine* (308 publications, IF: 2.9) published the most papers on cervical spondylosis surgery, followed by *European Spine Journal* (141 publications, IF: 2.5) and *Journal of Neurosurgery Spine* (119 publications, IF: 3.0). These top three productive journals published more than one fourth of all regarding cervical spondylosis surgery, while *Spine* published almost one sixth of all the studies. The top ten productive journals published 996 papers regarding cervical spondylosis surgery, accounting for almost half of all publications (Fig. 5).

### Authors of Publications

**Fig. 6:**
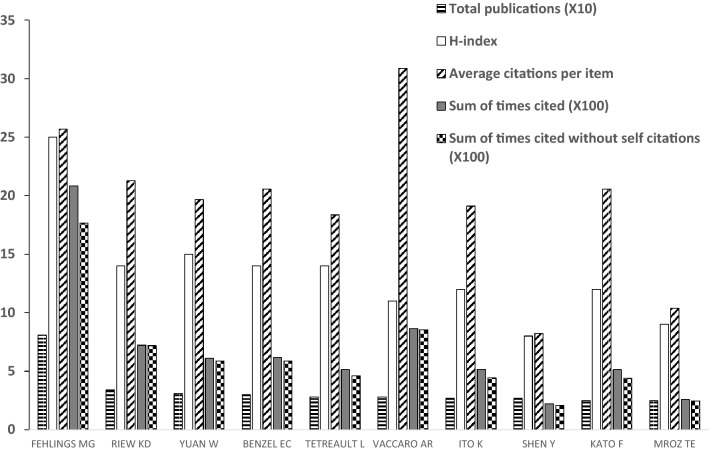
The total publications, H-index, average citations and sum of times cited of some productive authors

84 authors published more than ten publications on cervical spondylosis surgery, and 18 authors contributed more than twenty publications. Three authors published more than 30 publications. Fehlings MG is the most productive author with 81 papers, followed by Riew KD (34 publications) and Yuan W (31 publications). Fehlings MG (25) ranked first in H-index, followed by Yuan W (15), Riew KD (14), Benzel EC (14) and Tetreault L (14). Top three authors in average citations are Vaccaro AR (30.86), Fehlings MG (25.69) and Riew KD (21.26). Kato F tied for ninth in total publications, while ranked fifth in average citations. Ito K and Shen Y published the same number of studies related to cervical spondylosis surgery, while total citation number of publications of Ito K is about 2.3 times as that of Shen Y. Kato F and Mroz TE published the same number of publications on cervical spondylosis surgery, while sum of times cited of publications of Ito K is almost twice as that of Mroz TE (Fig. 6).

### Bibliometric Maps of Co-citations

One of the most important methods of bibliometric analysis is co-citations of references analysis. Due to the large number of cited references, the minimum number of citations of a cited reference was set as 50. Of the 22,939 cited references, 104 references were found to meet the threshold and chose for analyzing.

**Fig. 7:**
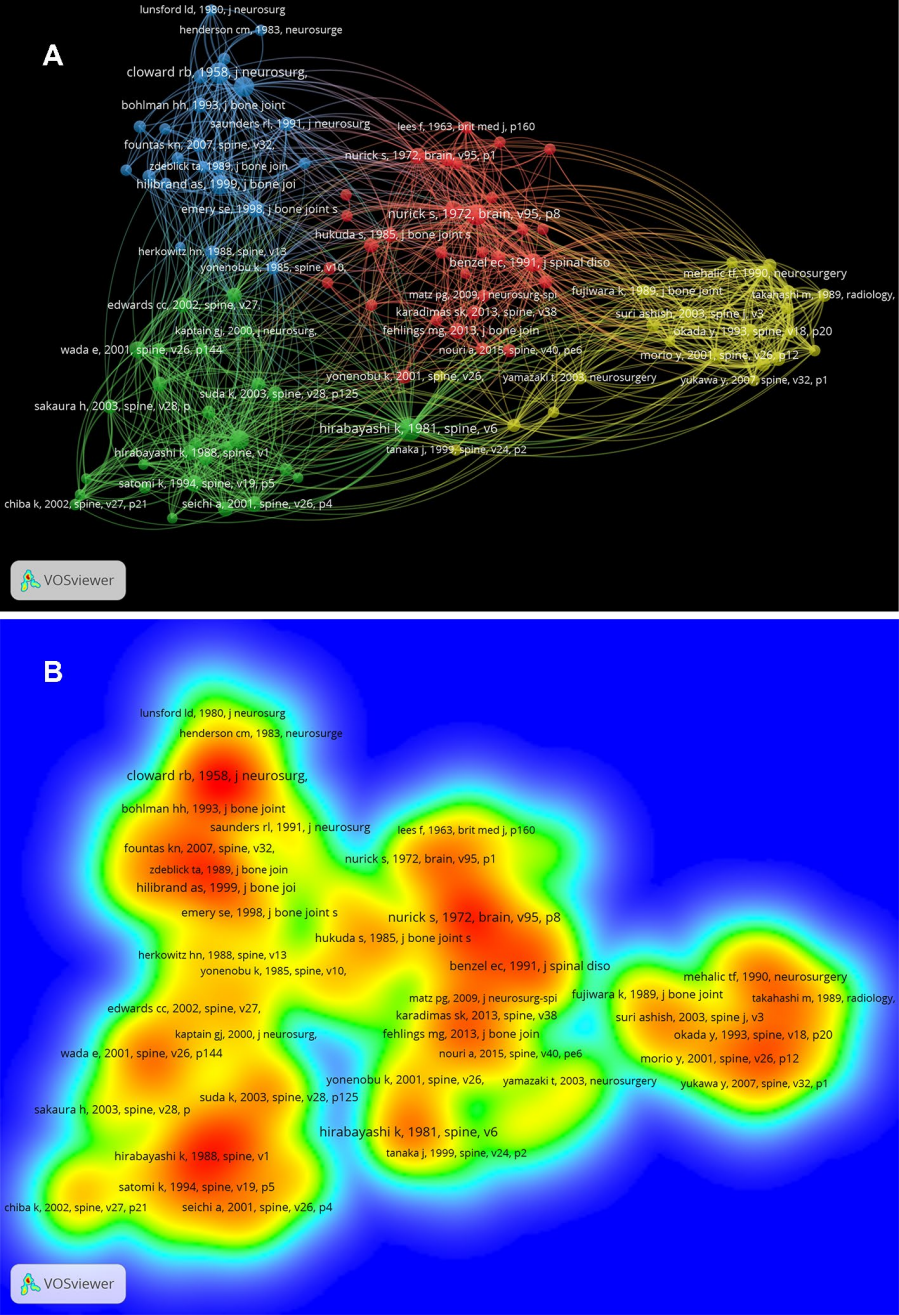
**a** Mapping on co-cited references of studies related to cervical spondylosis surgery. The 104 points with different colors represent the 104 cited references. Different colors of points divided the publications into different clusters. The size of a point represents the citation number of the publication. The line between two points represents that both publications had been cited in one paper. The length of a line represents the closeness of the two publications; the link is closer, the length of the line is shorter. **b** Mapping on density visualization of co-cited references. Different colors indicate different citation frequency. Blue represents few times, green represents average times and red represents many times. Items in one red circle linked closer with each other than that in other color areas

For each of the 104 cited references, the co-citation links with other cited references were analyzed and visualized by VOSviewer software (Fig. 7). The first cluster included 31 publications and mainly focused on natural history of cervical spondylosis. The second cluster included 28 publications and mainly focused on posterior approach surgery. The third cluster included 23 publications and mainly focused on anterior approach surgery. The fourth cluster included 22 publications and mainly focused on MRI of cervical spondylosis.

**Fig. 8:**
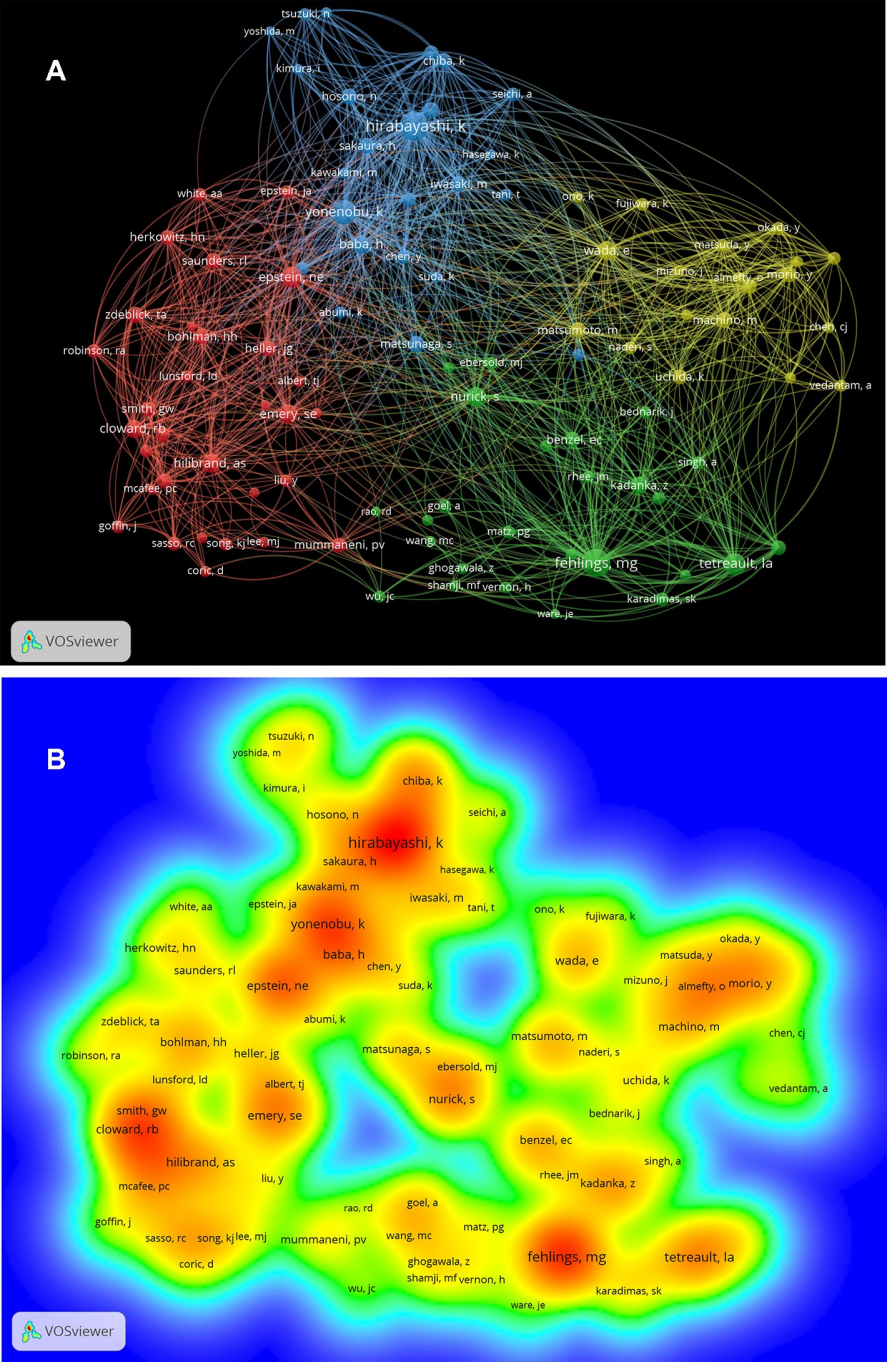
**a** Mapping on co-cited authors of papers of cervical spondylosis surgery. The 102 points with different colors represent the 102 cited authors. Different colors of points mean that the authors are divided into different clusters. The bigger size of a point means more citation number of the author. The line between two points represents that both authors had been cited in one publication. The length of a line represents the closeness of the two authors; shorter line means the link is closer. **b** Mapping on density visualization of co-cited authors. Different colors indicate different citation frequency. Blue represents few times, green represents average times and red represents many times. Items in one red circle linked closer with each other than that in other color areas

Co-citations of authors was also analyzed by VOSviewer software. Considering the large number of cited authors, the minimum number of citations of an author was set as 80. Of the 14,864 authors, 102 authors had been found to meet the threshold and selected for analyzing (Fig. 8).

### Hotspots of Papers on Cervical Spondylosis Surgery

**Fig. 9:**
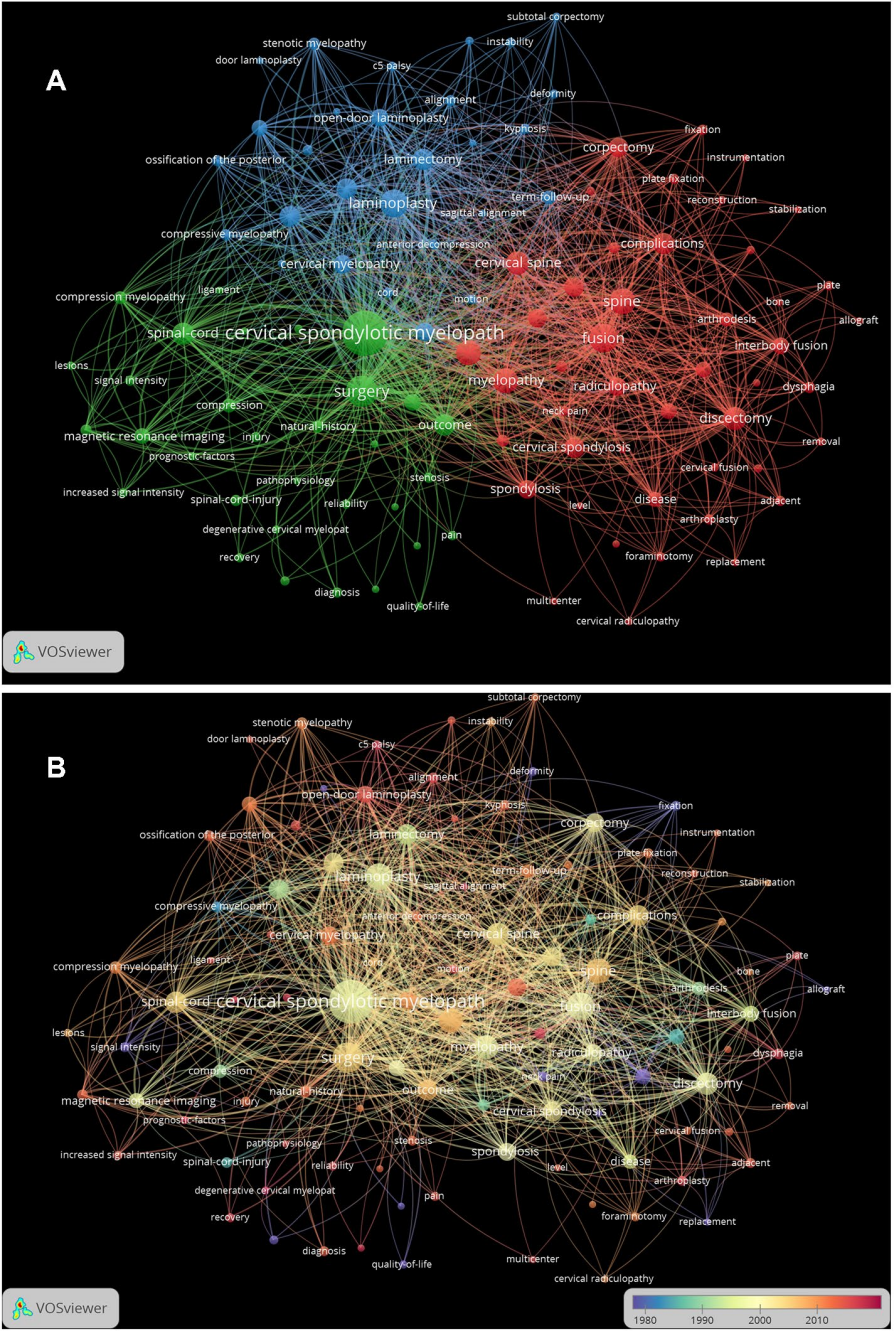
**a** Mapping on co-occurrence of keywords related to cervical spondylosis surgery. The 106 points with different colors represent the 106 keywords. The size of a point represents the frequency of the keywords. The line between two points represents that both keywords occurred in one paper. **b** Visualization of time when a keyword appeared. Keywords in purple appeared earlier than that in red

Figure 9 shows the keywords analysis of publications regarding cervical spondylosis surgery by VOSviewer. Due to the large number of keywords, the minimum number of occurrences of a keyword was set as 30, of the 5187 keywords, 106 met the threshold and chose for analyzing. The keywords were classified into three clusters. Red points (cluster 1) which consist of 46 items are mainly about ACDF and ACCF. Green points (cluster 2) which consist of 32 items are mainly about cervical spondylotic myelopathy. Blue points (cluster 3) which consist of 28 items are mainly about laminoplasty and laminectomy. “Degenerative cervical myelopathy” is the latest keyword that appeared in 2017 in 42 publications. “Surgical outcomes”, “association”, “sagittal alignment” and “prognostic-factors” are other relatively recent keywords appeared in 2016 in 33, 31, 34 and 37 publications, respectively. Table 1 shows publications of top 20 total citation frequency. Table 2 shows publications of top 20 average citation frequency per year. Table 3 shows keywords of top 20 occurrence frequency.

**Table 1:** Publications of top 20 total citation frequency

Title	Authors	Source title	Publication year	Total citations	Average per year
Epidemiology of cervical radiculopathy—a population-based study from Rochester, Minnesota, 1976 through 1990	Radhakrishnan, K et al	Brain	1994	439	16.26
Anterior cervical discectomy and fusion-associated complications	Fountas, Kostas N et al	Spine	2007	423	30.21
Anterior cervical decompression and arthrodesis for the treatment of cervical spondylotic myelopathy—2–17-year follow-up	Emery, SE et al	Journal of Bone and Joint Surgery-American Volume	1998	269	11.7
Neck and shoulder pain after laminoplasty—a noticeable complication	Hosono, N et al	Spine	1996	245	9.8
Kyphotic malalignment after anterior cervical fusion is one of the factors promoting the degenerative process in adjacent intervertebral levels	Katsuura, A et al	European Spine Journal	2001	244	12.2
Long-term follow-up-studies of open-door expansive laminoplasty for cervical stenotic myelopathy	Satomi, K et al	Spine	1994	240	8.89
Interobserver and intraobserver reliability of the Japanese Orthopaedic Association scoring system for evaluation of cervical compression myelopathy	Yonenobu, K et al	Spine	2001	228	11.4
C5 palsy after decompression surgery for cervical myelopathy—review of the literature	Sakaura, H et al	Spine	2003	218	12.11
Long-term results of double-door laminoplasty for cervical stenotic myelopathy	Seichi, A et al	Spine	2001	217	10.85
Long-term results of expansive open-door laminoplasty for cervical myelopathy—average 14-year follow-up study	Chiba, Kazuhiro et al	Spine	2006	216	14.4
Subtotal corpectomy versus laminoplasty for multilevel cervical spondylotic myelopathy—a long-term follow-up study over 10 years	Wada, E et al	Spine	2001	210	10.5
Prospective, randomized, multicenter study of cervical arthroplasty: 269 patients from the kineflexic artificial disc investigational device exemption study with a minimum 2-year follow-up Clinical article	Coric, Domagoj et al	Journal of Neurosurgery-Spine	2011	206	20.6
Local kyphosis reduces surgical outcomes of expansive open-door laminoplasty for cervical spondylotic myelopathy	Suda, K et al	Spine	2003	206	11.44
Cervical Radiographical Alignment Comprehensive Assessment Techniques and Potential Importance in Cervical Myelopathy	Ames, Christopher P et al	Spine	2013	187	23.38
Long-term results of expansive laminoplasty for ossification of the posterior longitudinal ligament of the cervical spine: more than 10 years follow-up	Iwasaki, M et al	Journal Of Neurosurgery	2002	184	9.68
Complications and mortality associated with cervical spine surgery for degenerative disease in the United States	Wang, Marjorie C et al	Spine	2007	183	13.07
Incidence and outcome of kyphotic deformity following laminectomy for cervical spondylotic myelopathy	Kaptain, GJ et al	Journal Of Neurosurgery	2000	179	8.52
Laminoplasty versus laminectomy and fusion for multilevel cervical myelopathy—an independent matched cohort analysis	Heller, JG et al	Spine	2001	178	8.9
Surgical treatment for cervical spondylotic myelopathy	Ebersold, MJ et al	Journal Of Neurosurgery	1995	178	6.85
Neurologic complications of surgery for cervical compression myelopathy	Yonenobu, K et al	Spine	1991	173	5.77

**Table 2:** Publications of top 20 average citation frequency per year

Title	Authors	Source title	Publication year	Total citations	Average per year
Anterior cervical discectomy and fusion-associated complications	Fountas, Kostas N et al	Spine	2007	423	30.21
Cervical radiographical alignment comprehensive assessment techniques and potential importance in cervical myelopathy	Ames, Christopher P et al	Spine	2013	187	23.38
Prospective, randomized, multicenter study of cervical arthroplasty: 269 patients from the kineflexic artificial disc investigational device exemption study with a minimum 2-year follow-up clinical article	Coric, Domagoj et al	Journal of Neurosurgery-Spine	2011	206	20.6
Efficacy and safety of surgical decompression in patients with cervical spondylotic myelopathy results of the AOSpine North America prospective multicenter study	Fehlings, Michael G et al	Journal of Bone and Joint Surgery-American Volume	2013	159	19.88
Cervical spondylotic myelopathy: the clinical phenomenon and the current pathobiology of an increasingly prevalent and devastating disorder	Kalsi-Ryan, Sukhvinder et al	Neuroscientist	2013	147	18.38
Epidemiology of cervical radiculopathy—a population-based study from Rochester, Minnesota, 1976 through 1990	Radhakrishnan, K et al	Brain	1994	439	16.26
Epidemiology, diagnosis, and treatment of neck pain	Cohen, Steven P	Mayo Clinic Proceedings	2015	92	15.33
Results of cervical arthroplasty compared with anterior discectomy and fusion: 4-year clinical outcomes in a prospective, randomized controlled trial	Sasso, Rick C et al	Journal of Bone And Joint Surgery-American Volume	2011	150	15
Long-term results of expansive open-door laminoplasty for cervical myelopathy—average 14-year follow-up study	Chiba, Kazuhiro et al	Spine	2006	216	14.4
Perioperative and delayed complications associated with the surgical treatment of cervical spondylotic myelopathy based on 302 patients from the AOSpine North America cervical spondylotic myelopathy study presented at the 2011 spine section meeting clinical article	Fehlings, Michael G et al	Journal of Neurosurgery-Spine	2012	118	13.11
Complications and mortality associated with cervical spine surgery for degenerative disease in the united states	Wang, Marjorie C et al	Spine	2007	183	13.07
A global perspective on the outcomes of surgical decompression in patients with cervical spondylotic myelopathy	Fehlings, Michael G et al	Spine	2015	77	12.83
A predictive model of complications after spine surgery: the national surgical quality improvement program (nsqip) 2005–2010	Bekelis, Kimon et al	Spine Journal	2014	89	12.71
C5 palsy after cervical laminoplasty a multicentre study	Imagama, S et al	Journal of Bone And Joint Surgery-British Volume	2010	139	12.64
Complications in spine surgery a review	Nasser, Rani et al	Journal of Neurosurgery-Spine	2010	138	12.55
Cervical spondylotic myelopathy	Tracy, Jennifer A et al	Neurologist	2010	137	12.45
Kyphotic malalignment after anterior cervical fusion is one of the factors promoting the degenerative process in adjacent intervertebral levels	Katsuura, A et al	European Spine Journal	2001	244	12.2
C5 palsy after decompression surgery for cervical myelopathy—review of the literature	Sakaura, H et al	Spine	2003	218	12.11
Anterior versus posterior surgical approaches to treat cervical spondylotic myelopathy outcomes of the prospective multicenter AOSpine North America CSM study in 264 patients	Fehlings, Michael G et al	Spine	2013	94	11.75
Anterior cervical decompression and arthrodesis for the treatment of cervical spondylotic myelopathy—2–17-year follow-up	Emery, SE et al	Journal of Bone And Joint Surgery-American Volume	1998	269	11.7

**Table 3:** Keywords of top 20 occurrence frequency

Label	Cluster	Weight < Occurrences >	Weight < Total link strength >	Score < Avg. pub. year >	Score < Avg. citations >
Cervical spondylotic myelopathy	2	1088	6021	1997.9256	18.0441
Surgery	2	542	3207	2004.1181	20.6993
Fusion	1	459	2964	1999.3529	17.7952
Laminoplasty	3	425	2879	1998.0988	19.2565
Spine	1	366	2260	2006.1202	19.4262
Myelopathy	1	353	2079	1998.5156	20.7224
Decompression	1	330	2298	2006.7545	19.4242
Discectomy	1	298	1845	1998.4228	21.6174
Cervical spine	1	295	1819	2003.0508	24.1559
Spinal cord	2	278	1670	2004.0252	24.9281
Outcome	2	251	1676	2006.498	16.247
Complications	1	246	1647	2004.2317	22.748
Laminectomy	3	244	1832	1994.623	23
Cervical spondylosis	1	235	1142	2001.5447	20.6894
Ossification	3	226	1641	2002.1637	23.4336
Posterior longitudinal ligament	3	225	1576	1993.0444	22.4044
Follow-up	1	213	1459	2002.3286	20.8263
Corpectomy	1	203	1438	2000.9951	27.335
Spondylosis	1	196	1002	1999.5306	18.6735
Radiculopathy	1	192	1245	1998.9479	27.0781

## Discussion

### Trends of Publications on Cervical Spondylosis Surgery

Roughly growing trends have been noticed both in published papers and relative research interest in the last 3 decades, showing that cervical spondylosis surgery is a hotspot domain of spine surgery.

Top three countries/regions account for more than 70% of all the publications regarding cervical spondylosis surgery, indicating that publications related to this domain are very concentrated. USA ranked first both in total publication number and H-index, and ranked second in average citation times, indicating that USA dominates studies in the domain of cervical spondylosis surgery. The publication number of England ranked only eighth; however, the average citations of it ranked first of all the countries/regions, indicating that the quality of publications from England is relatively high and may pay more attention on the quantity of the publications. The publication number and H-index of China (not include Taiwan region) ranked third and fourth, respectively, of all the countries/regions, while the average citations of it ranked only tenth among the top ten productive countries/regions which suggested that China (not include Taiwan region) may improve the quality instead of only focusing on the quantity of the publications regarding cervical spondylosis surgery. University of Toronto, PLA Second Military Medical University and Jefferson University are some of the most productive organizations related to the publications of cervical spondylosis surgery. PLA Second Military Medical University ranked the second most productive organizations, while the average citations only ranked ninth of all the top ten productive organizations, indicating that it may focus more on the quality instead of quantity regarding studies of cervical spondylosis surgery. Case Western Reserve University and Hebei Medical University published the same number of studies regarding cervical spondylosis surgery, while the total sum of times cited of papers of Case Western Reserve University is more than three times as that of Hebei Medical University, suggesting that quality of papers related to cervical spondylosis surgery of Hebei Medical University is relatively low and should pay more attention to. Future studies regarding cervical spondylosis surgery are most likely originated from these aforementioned organizations, and scholars in this domain are recommended to collaborate with researchers in these organizations. Publications regarding cervical spondylosis surgery are concentrated in certain journals. The top three productive journals published more than one fourth papers of all, and the top ten productive journals published almost half of all publications, suggesting that future publications in this domain are most likely to be published on the aforementioned journals, studies of cervical spondylosis surgery are recommended to submit to these journals. Fehlings MG both ranked first in publication number and H-index, and ranked second in average citations, suggesting that this author is best in studies of this field. The research quality of Shen Y is relatively lower than that of Ito K and the research quality of Mroz TE is relatively lower than that of Kato F, indicating that Shen Y and Mroz TE may pay more attention to improve the quality of their studies on cervical spondylosis surgery.

In visualized maps of co-citations of authors, the size of a point means the citation number of the author and the shorter length of the line means the link is closer. Researchers in this domain could cooperate with others whose points are big and near. Scholars may cooperate with other authors whose points are big and distant if they would like to expand other research areas.

In visualized maps of co-occurrence of keywords, the size of a point indicates the frequency of the keyword. The line between two points represents that the two keywords appeared in one paper. For the sake of discovering new idea in this domain, scholars may find big and distant points besides their own keywords.

### Studies Focused on Cervical Spondylosis Surgery

Radhakrishnan, K’s article titled “Epidemiology of cervical radiculopathy. A population-based study from Rochester, Minnesota, 1976 through 1990” published in *Brain* back in 1994 ranked first in total citations (439 times). This study showed the epidemiology description of cervical radiculopathy in Rochester. C7 is the most common involved nerve root in monoradiculopathy, followed by C6. 26% of all underwent surgery treatment for cervical radiculopathy. For total population in Rochester, the average annual age-adjusted incidence of cervical radiculopathy was 83.2/100,000, while 107.3/100,000 for males and 63.5/100,000 for females [4].

Article titled “Anterior cervical discectomy and fusion associated complications” ranked first in average citation frequency per year (30.21 times per year) which is published in *Spine* back in 2007 by Fountas, KN. This article is also ranked second in the total citation number (423 times) of all the publications related to this field. Rate of complication occurrence of ACDF is generally underestimated, and data of the real incidence with large cases are still lacking. This retrospective study included 1015 patients and found that overall complication rate was 19.3%. The most common (9.5%) complication was dysphagia. 5.6% of patients suffered postoperative hematoma, while 2.4% required surgical intervention. Other complication rates are as follows: dural penetration (5‰), esophageal perforation (3‰), preexisting myelopathy deterioration (2‰). The rates of Horner’s syndrome, instrumentation backout and superficial wound infection are all 1‰ [21].

*Spin*e published an article in 2013 by Ames, CP, named “Cervical radiographical alignment comprehensive assessment techniques and potential importance in cervical myelopathy” ranked second in average citation frequency per year (23.38 times per year). This narrative review summarized multiple cervical alignment parameters and their association with cervical myelopathy and deformity. Authors indicated that cervical sagittal alignment is related to thoracolumbar spinal pelvic alignment and with T1 slope, and also related to reginal disability and severity of myelopathy. This study emphasized cervical kyphosis and cervical sagittal imbalance, if any, should be considered to correct in the decompressive surgery [22].

*Journal of Neurosurgery-Spine* published an article in 2011 by Coric, D, titled “Prospective, randomized, multicenter study of cervical arthroplasty: 269 patients from the Kineflex|C artificial disc investigational device exemption study with a minimum 2-year follow-up: clinical article” ranked third in average citation frequency per year (20.6 times per year). This prospective, multiple center, randomized clinical trial compared cervical total disc replacement (CTDR) and anterior cervical discectomy and fusion (ACDF) in treating single-level cervical spondylosis radiculopathy with more than 2-year follow-up. This study indicated that both procedures are effective and not significantly different in blood loss, reoperation rate, length of hospital stay and surgical time. CTDR is able to maintain better motion than ACDF, and significantly fewer severe radiographic changes of adjacent level are shown in the CTDR [23].

### Strengths and Limitations

Multiple bibliometric methods were used to analyze publications related to cervical spondylosis surgery from Web of Science database. This study also has some limitations as follows. Bibliometric analysis maybe not always reflects the actual situation, while newly published publications are not cited much times because of short publication time. Some of non-English publications are not included in this study that may lead to inaccurate conclusion.

## Conclusion

In conclusion, this bibliometric analysis indicated that roughly growing trends have been noticed not only in published papers but also in relative research interest in the last 3 decades, showing that cervical spondylosis surgery is a hotspot domain of spine surgery. USA dominates the research related to cervical spondylosis surgery. The quality of publications from England is relatively high and may pay more attention on the quantity of the publications, while China (not include Taiwan region) may improve the quality instead of only focusing on the quantity of the publications. University of Toronto, PLA Second Military Medical University and Jefferson University are some of the most productive organizations related to the publications of cervical spondylosis surgery. *Spine, European Spine Journal* and *Journal of Neurosurgery Spine* are the top three productive journals on publications of cervical spondylosis surgery. Fehlings MG is best in studies of this field. “Degenerative cervical myelopathy”, “surgical outcomes”, “association”, “sagittal alignment” and “prognostic-factors” may be the new research hotspots in this field.
